# Is ankyloglossia associated with obstructive sleep apnea?^☆^

**DOI:** 10.1016/j.bjorl.2021.09.008

**Published:** 2021-11-05

**Authors:** Marieli Timpani Bussi, Camila de Castro Corrêa, Arthur Justi Cassettari, Lorena Torres Giacomin, Ana Célia Faria, Ana Paula Sereni Manfredi Moreira, Itamá Magalhães, Mila Oliveira da Cunha, Silke Anna Theresa Weber, Edilson Zancanella, Almiro José Machado Júnior

**Affiliations:** a Universidade Estadual de Campinas (UNICAMP), Departamento de Otorrinolaringologia Cabeça e Pescoço, Campinas, SP, Brazil; b Centro Universitário Planalto do Distrito Federal (UNIPLAN), Brasília, DF, Brazil; c Universidade de Brasília (UnB), Brasília, DF, Brazil; d Universidade Estadual Paulista Júlio de Mesquita Filho (UNESP), Faculdade de Medicina, Botucatu, SP, Brazil

**Keywords:** Ankyloglossia, Obstructive sleep apnea, Myofunctional therapy

## Abstract

**Objectives:**

To investigate the evidence on the association between ankyloglossia and obstructive sleep apnea.

**Methods:**

An integrative literature review was carried out in the databases. Observational and interventional studies that assessed the lingual frenulum in children with sleep-disordered breathing were included. As exclusion criteria: studies in animals, *in vitro*, letters to the editor, expert opinions, other reviews. The selected articles were analyzed regarding the study design, sample, characterization of the lingual frenulum and sleep assessment, in addition to the main results and conclusions.

**Results:**

Ninety-seven articles were identified, but only 4 met the inclusion criteria. Two retrospective studies concluded that the untreated shortened lingual frenulum at birth is associated with obstructive sleep apnea. A prospective study concluded that there was an improvement in speech and deglutition after lingual frenectomy, in addition to improved sleep. A retrospective cohort concluded that lingual frenuloplasty combined with myofunctional therapy is effective in the treatment of snoring and mouth breathing.

**Conclusion:**

The studies included in this review contribute to corroborate the association between ankyloglossia and obstructive sleep apnea.

## Highlights

• An untreated shortened lingual frenulum at birth is associated with sleep apnea.
• A shortened lingual frenulum alters the craniofacial growth with respiratory consequences.
• Lingual frenuloplasty associated with myofunctional therapy is effective in the treatment of snoring.
• In addition to improved sleep, there was an improvement in the speech and deglutition functions.

## Introduction

Obstructive sleep apnea (OSA) is a respiratory disorder characterized by recurrent airway obstruction during sleep, and its causes are multifactorial.^1^ The population prevalence is high, and cardiovascular alterations are among its consequences. Treatment is sometimes multidisciplinary, with the continuous positive airway pressure (CPAP) device being the gold standard.^2^, ^3^, ^4^ To understand the pathology, we need to understand that the causes are varied and, therefore, the treatment must be individualized.

Among the anatomical findings of OSA, one can cite septal deviation, palatine tonsil hypertrophy, transverse maxillary atresia, maxillomandibular retrusion, or even a combination of them.^3^ Other phenotypes were defined as: (1) anatomically compromised upper airways (UAs) (pharyngeal critical closing pressure (Pcrit) (2) Inappropriate responsiveness of the UA dilator muscles during sleep, (3) low arousal threshold (4) unstable ventilatory control (high loop gain).^3^, ^4^, ^5^

Ankyloglossia is considered a congenital anomaly found in 4%–5% of the general population.^6^ It can be inherited as an X-linked autosomal dominant trait, being more common in males. The presence of a shortened lingual frenulum has been reported in genetically-related syndromes such as Beckwith-Wiedemann, digital-orofacial syndrome, cleft palate and Opitz syndrome, but all mutations associated with these syndromes are not known. It is possible that the shortened lingual frenulum is associated with tongue malposition due to an unknown mutation, but there is no evidence to support this hypothesis.^7^, ^8^, ^9^

A shortened lingual frenulum has been shown to lead to mouth breathing and abnormal development of the oral cavity,^10^, ^11^, ^12^ increasing the risk of upper airway (UA) collapsibility during sleep.

The interaction between bone growth stimulation and the absence of nasal breathing can lead to an abnormal orofacial development and reduction in the ideal size of the UAs, causing abnormal breathing during sleep over time, initially with flow limitation and then with progressive worsening of obstructive events, leading to Obstructive Sleep Apnea.

Therefore, there is evidence of an association between the presence of a shortened lingual frenulum and changes in craniofacial growth, with possible consequences on the caliber of the upper airways.

Ankyloglossia limits the mobility of the tongue,^13^ impairing the sucking, chewing, deglutition and speaking functions. With the alteration of the stomatognathic functions, the stability of the structure is not maintained.^14^

When changes in the lingual frenulum are not treated early, they can compromise breastfeeding, which plays an important role in the development of bone structures and maintenance of nasal breathing.^13^, ^14^

Several studies have reported that a shortened lingual frenulum can lead to changes in craniofacial growth, with respiratory consequences, including obstructive sleep apnea.^15^, ^16^, ^17^

## Objective

To investigate the evidence on the association between ankyloglossia and obstructive sleep apnea.

## Methods

An integrative literature review was carried out in the following databases: Pubmed, Scopus, Embase, Web of Science, Lilacs and ProQuest. The PECOS criteria were employed to structure the search strategy (P = Participants; E = Exposure; C = Comparison; O = Outcomes, S = Types of Study), shown in Table 1.

**Table 1:** PECOs criteria for the research strategy of the present study.

PECOS	
***P****articipants*	Population
***E****xposition*	Obstructive sleep apnea
***C****omparison*	No comparison
***O****utcomes*	Studies that performed the assessment of the lingual frenulum.
*Types of****S****tudies*	Observational and interventional studies

The keywords and free terms used corresponded to the established PECOs (Table 2). Observational and interventional studies that assessed the lingual frenulum in children with sleep-disordered breathing were included. As exclusion criteria, studies in animals, *in vitro*, letters to the editor, expert opinions, and other reviews (secondary studies) were excluded. There was no limitation related to language or year of publication.

**Table 2:** Search performed in the databases and the respective initial results.

Database	Search	Initial findings
Pubmed	(“Sleep Apnea, Obstructive” [MeSH Terms] OR “Apneas, Obstructive Sleep” OR “Obstructive Sleep Apneas” OR “Sleep Apneas, Obstructive” OR “Obstructive Sleep Apnea Syndrome” OR “Obstructive Sleep Apnea” OR “OSAHS” OR “Syndrome, Sleep Apnea, Obstructive” OR “Sleep Apnea Syndrome, Obstructive” OR “Apnea, Obstructive Sleep” OR “Sleep Apnea Hypopnea Syndrome” OR “Syndrome, Obstructive Sleep Apnea” OR “Upper Airway Resistance Sleep Apnea Syndrome” OR “Syndrome, Upper Airway Resistance, Sleep Apnea” OR “OSA” OR “OSAS” OR “Sleep Apnea Syndromes” [MeSH Terms] OR “Apnea Syndrome, Sleep” OR “Apnea Syndromes, Sleep” OR “Sleep Apnea Syndrome” OR “Sleep Hypopnea” OR “Hypopnea, Sleep” OR “Hypopneas, Sleep” OR “Sleep Hypopneas” OR “Apnea, Sleep” OR “Apneas, Sleep” OR “Sleep Apnea” OR “Sleep Apneas” OR “Sleep Apnea, Mixed Central and Obstructive” OR “Mixed Central and Obstructive Sleep Apnea” OR “Sleep Apnea, Mixed” OR “Mixed Sleep Apnea” OR “Mixed Sleep Apneas” OR “Sleep Apneas, Mixed” OR “Hypersomnia with Periodic Respiration” OR “Sleep-Disordered Breathing” OR “Breathing, Sleep-Disordered” OR “Sleep Disordered Breathing”) AND (“Lingual Frenum” [MeSH Terms] OR “Lingual Frenulum” OR “Frenulum, Lingual” OR “Frenulums, Lingual” OR “Lingual Frenulums” OR “Frenum, Lingual” OR “Frenums, Lingual” OR “Lingual Frenums”)	10
Scopus	(“Sleep Apnea, Obstructive” OR “Apneas, Obstructive Sleep” OR “Obstructive Sleep Apneas” OR “Sleep Apneas, Obstructive” OR “Obstructive Sleep Apnea Syndrome” OR “Obstructive Sleep Apnea” OR “OSAHS” OR “Syndrome, Sleep Apnea, Obstructive” OR “Sleep Apnea Syndrome, Obstructive” OR “Apnea, Obstructive Sleep” OR “Sleep Apnea Hypopnea Syndrome” OR “Syndrome, Obstructive Sleep Apnea” OR “Upper Airway Resistance Sleep Apnea Syndrome” OR “Syndrome, Upper Airway Resistance, Sleep Apnea” OR “OSA” OR “OSAS” OR “Sleep Apnea Syndromes” OR “Apnea Syndrome, Sleep” OR “Apnea Syndromes, Sleep” OR “Sleep Apnea Syndrome” OR “Sleep Hypopnea” OR “Hypopnea, Sleep” OR “Hypopneas, Sleep” OR “Sleep Hypopneas” OR “Apnea, Sleep” OR “Apneas, Sleep” OR “Sleep Apnea” OR “Sleep Apneas” OR “Sleep Apnea, Mixed Central and Obstructive” OR “Mixed Central and Obstructive Sleep Apnea” OR “Sleep Apnea, Mixed” OR “Mixed Sleep Apnea” OR “Mixed Sleep Apneas” OR “Sleep Apneas, Mixed” OR “Hypersomnia with Periodic Respiration” OR “Sleep-Disordered Breathing” OR “Breathing, Sleep-Disordered” OR “Sleep Disordered Breathing”) AND (“Lingual Frenum” OR “Lingual Frenulum” OR “Frenulum, Lingual” OR “Frenulums, Lingual” OR “Lingual Frenulums” OR “Frenum, Lingual” OR “Frenums, Lingual” OR “Lingual Frenums”)	45
Embase	(‘sleep apnea, obstructive’/exp OR ‘sleep apnea, obstructive’ OR ‘apneas, obstructive sleep’ OR ‘obstructive sleep apneas’ OR ‘sleep apneas, obstructive’ OR ‘obstructive sleep apnea syndrome’/exp OR ‘obstructive sleep apnea syndrome’ OR ‘obstructive sleep apnea’/exp OR ‘obstructive sleep apnea’ OR ‘osahs’ OR ‘syndrome, sleep apnea, obstructive’ OR ‘sleep apnea syndrome, obstructive’ OR ‘apnea, obstructive sleep’ OR ‘sleep apnea hypopnea syndrome’/exp OR ‘sleep apnea hypopnea syndrome’ OR ‘syndrome, obstructive sleep apnea’ OR ‘upper airway resistance sleep apnea syndrome’ OR ‘syndrome, upper airway resistance, sleep apnea’ OR ‘osa’ OR ‘osas’ OR ‘sleep apnea syndromes’/exp OR ‘sleep apnea syndromes’ OR ‘apnea syndrome, sleep’ OR ‘apnea syndromes, sleep’ OR ‘sleep apnea syndrome’/exp OR ‘sleep apnea syndrome’ OR ‘sleep hypopnea’ OR ‘hypopnea, sleep’ OR ‘hypopneas, sleep' OR ‘sleep hypopneas’ OR ‘apnea, sleep’/exp OR ‘apnea, sleep’ OR ‘apneas, sleep’ OR ‘sleep apnea’/exp OR ‘sleep apnea’ OR ‘sleep apneas’ OR ‘sleep apnea, mixed central and obstructive’ OR ‘mixed central and obstructive sleep apnea’ OR ‘sleep apnea, mixed’ OR ‘mixed sleep apnea’ OR ‘mixed sleep apneas’ OR ‘sleep apneas, mixed’ OR ‘hypersomnia with periodic respiration’ OR ‘sleep-disordered breathing’/exp OR ‘sleep-disordered breathing’ OR ‘breathing, sleep-disordered’ OR ‘sleep disordered breathing’/exp OR ‘sleep disordered breathing’) AND (‘lingual frenum’/exp OR ‘lingual frenum’ OR ‘lingual frenulum’/exp OR ‘lingual frenulum’ OR ‘frenulum, lingual’/exp OR ‘frenulum, lingual’ OR ‘frenulums, lingual’ OR ‘lingual frenulums’ OR ‘frenum, lingual’ OR ‘frenums, lingual’ OR ‘lingual frenums’)	20
Web of Science	(“Sleep Apnea, Obstructive” OR “Apneas, Obstructive Sleep” OR “Obstructive Sleep Apneas” OR “Sleep Apneas, Obstructive” OR “Obstructive Sleep Apnea Syndrome” OR “Obstructive Sleep Apnea” OR “OSAHS” OR “Syndrome, Sleep Apnea, Obstructive” OR “Sleep Apnea Syndrome, Obstructive” OR “Apnea, Obstructive Sleep” OR “Sleep Apnea Hypopnea Syndrome” OR “Syndrome, Obstructive Sleep Apnea” OR “Upper Airway Resistance Sleep Apnea Syndrome” OR “Syndrome, Upper Airway Resistance, Sleep Apnea” OR “OSA” OR “OSAS” OR “Sleep Apnea Syndromes” OR “Apnea Syndrome, Sleep” OR “Apnea Syndromes, Sleep” OR “Sleep Apnea Syndrome” OR “Sleep Hypopnea” OR “Hypopnea, Sleep” OR “Hypopneas, Sleep” OR “Sleep Hypopneas” OR “Apnea, Sleep” OR “Apneas, Sleep” OR “Sleep Apnea” OR “Sleep Apneas” OR “Sleep Apnea, Mixed Central and Obstructive” OR “Mixed Central and Obstructive Sleep Apnea” OR “Sleep Apnea, Mixed” OR “Mixed Sleep Apnea” OR “Mixed Sleep Apneas” OR “Sleep Apneas, Mixed” OR “Hypersomnia with Periodic Respiration” OR “Sleep-Disordered Breathing” OR “Breathing, Sleep-Disordered” OR “Sleep Disordered Breathing”) AND (“Lingual Frenum” OR “Lingual Frenulum” OR “Frenulum, Lingual” OR “Frenulums, Lingual” OR “Lingual Frenulums” OR “Frenum, Lingual” OR “Frenums, Lingual” OR “Lingual Frenums”)	9
Lilacs	(“Sleep Apnea, Obstructive” OR “Apneas, Obstructive Sleep” OR “Obstructive Sleep Apneas” OR “Sleep Apneas, Obstructive” OR “Obstructive Sleep Apnea Syndrome” OR “Obstructive Sleep Apnea” OR “OSAHS” OR “Syndrome, Sleep Apnea, Obstructive” OR “Sleep Apnea Syndrome, Obstructive” OR “Apnea, Obstructive Sleep” OR “Sleep Apnea Hypopnea Syndrome” OR “Syndrome, Obstructive Sleep Apnea” OR “Upper Airway Resistance Sleep Apnea Syndrome” OR “Syndrome, Upper Airway Resistance, Sleep Apnea” OR “OSA” OR “OSAS” OR “Sleep Apnea Syndromes” OR “Apnea Syndrome, Sleep” OR “Apnea Syndromes, Sleep” OR “Sleep Apnea Syndrome” OR “Sleep Hypopnea” OR “Hypopnea, Sleep” OR “Hypopneas, Sleep” OR “Sleep Hypopneas” OR “Apnea, Sleep” OR “Apneas, Sleep” OR “Sleep Apnea” OR “Sleep Apneas” OR “Sleep Apnea, Mixed Central and Obstructive” OR “Mixed Central and Obstructive Sleep Apnea” OR “Sleep Apnea, Mixed” OR “Mixed Sleep Apnea” OR “Mixed Sleep Apneas” OR “Sleep Apneas, Mixed” OR “Hypersomnia with Periodic Respiration” OR “Sleep-Disordered Breathing” OR “Breathing, Sleep-Disordered” OR “Sleep Disordered Breathing” OR “Apneia Obstrutiva do Sono” OR “Apneia do Sono Obstrutiva” OR “Apneia do Sono Tipo Obstrutiva” OR “Síndrome da Apneia Obstrutiva do Sono” OR “Síndrome de Apneia do Sono por Resistência das Vias Aéreas Superiores” OR “Apnea Obstructiva del Sueño” OR “Síndromes da Apneia do Sono” OR “Apneia do Sono” OR “Hipersonia com Respiração Periódica” OR “Respiração Desordenada Durante o Sono” OR “Síndromes de la Apnea del Sueño”) AND (“Lingual Frenum” OR “Lingual Frenulum” OR “Frenulum, Lingual” OR “Frenulums, Lingual” OR “Lingual Frenulums” OR “Frenum, Lingual” OR “Frenums, Lingual” OR “Lingual Frenums” OR “Freio Lingual” OR “Frenillo Lingual” OR “Frênulo da Língua”)	3
ProQuest	(“Sleep Apnea, Obstructive” OR “Apneas, Obstructive Sleep” OR “Obstructive Sleep Apneas” OR “Sleep Apneas, Obstructive” OR “Obstructive Sleep Apnea Syndrome” OR “Obstructive Sleep Apnea” OR “OSAHS” OR “Syndrome, Sleep Apnea, Obstructive” OR “Sleep Apnea Syndrome, Obstructive” OR “Apnea, Obstructive Sleep” OR “Sleep Apnea Hypopnea Syndrome” OR “Syndrome, Obstructive Sleep Apnea” OR “Upper Airway Resistance Sleep Apnea Syndrome” OR “Syndrome, Upper Airway Resistance, Sleep Apnea” OR “OSA” OR “OSAS” OR “Sleep Apnea Syndromes” OR “Apnea Syndrome, Sleep” OR “Apnea Syndromes, Sleep” OR “Sleep Apnea Syndrome” OR “Sleep Hypopnea” OR “Hypopnea, Sleep” OR “Hypopneas, Sleep” OR “Sleep Hypopneas” OR “Apnea, Sleep” OR “Apneas, Sleep” OR “Sleep Apnea” OR “Sleep Apneas” OR “Sleep Apnea, Mixed Central and Obstructive” OR “Mixed Central and Obstructive Sleep Apnea” OR “Sleep Apnea, Mixed” OR “Mixed Sleep Apnea” OR “Mixed Sleep Apneas” OR “Sleep Apneas, Mixed” OR “Hypersomnia with Periodic Respiration” OR “Sleep-Disordered Breathing” OR “Breathing, Sleep-Disordered” OR “Sleep Disordered Breathing” OR “Apneia Obstrutiva do Sono” OR “Apneia do Sono Obstrutiva” OR “Apneia do Sono Tipo Obstrutiva” OR “Síndrome da Apneia Obstrutiva do Sono” OR “Síndrome de Apneia do Sono por Resistência das Vias Aéreas Superiores” OR “Apnea Obstructiva del Sueño” OR “Síndromes da Apneia do Sono” OR “Apneia do Sono” OR “Hipersonia com Respiração Periódica” OR “Respiração Desordenada Durante o Sono” OR “Síndromes de la Apnea del Sueño”) AND (“Lingual Frenum” OR “Lingual Frenulum” OR “Frenulum, Lingual” OR “Frenulums, Lingual” OR “Lingual Frenulums” OR “Frenum, Lingual” OR “Frenums, Lingual” OR “Lingual Frenums” OR “Freio Lingual” OR “Frenillo Lingual” OR “Frênulo da Língua”)	10

The identified articles were exported to Endnote Web and, subsequently, to the Rayyan manager, where the first phase was carried out, with the reading of titles and abstracts. The reading and selection steps were performed by two reviewers, blindly. In case of conflicts, a third reviewer was called in. The articles included in the first phase were read in full for the final selection.

The selected articles were analyzed regarding the study design, sample, characterization of the lingual frenulum and sleep assessment, as well as the main results and conclusions.

The protocol registered in Prospero under number CRD42020206899.

## Results

**Figure 1:**
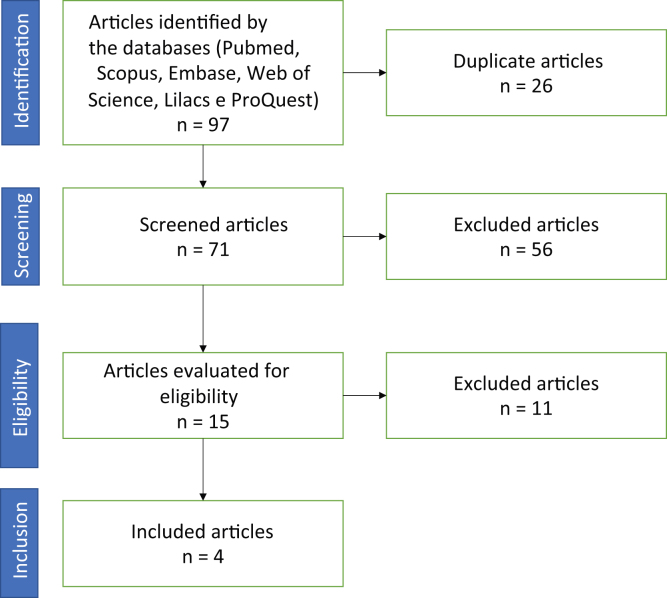
Flow diagram of the study selection.

A total of 97 articles were identified. After evaluating the duplicates, 16 articles were excluded. Thus, the reviewers read 71 titles and abstracts in the first phase. Of these, 10 were included by both and 6 showed conflicts. After the evaluation by the third reviewer, 15 articles were included for full reading. Of the 15 articles, 11 were excluded by the authors for not meeting the inclusion criteria or for having a study design that met the exclusion criteria. In the end, four articles were included in the study (Fig. 1).

The article by Guilleminault et al.,^18^ assessed 150 children aged 3–12 years in a retrospective study comparing children with a shortened lingual frenulum and children with a normal one. To measure the lingual frenulum, a measurement was taken during the maximum opening of the lower left incisor to the upper left incisor, and then a measurement was taken with the mouth wide open with the tip of the tongue touching the incisor papilla. This measure was considered normal if the difference between the two was <50%. There was a statistical significance between the Apnea/Hypopnea Index (AHI) of 13.06 ± 4.17 in the shortened lingual frenulum group *versus* 11.36 ± 5.39 in the normal lingual frenulum group (*p* = 0.025). The shortened lingual frenulum was predictive of a higher AHI in male (*p* = 0.0069), but not in female individuals (*p* = 0.8615). The study concluded that an untreated shortened lingual frenulum at birth is associated with OSAS at older age.

In another retrospective study, Villa et al.,^19^ assessed 504 children aged 6–14 years and evaluated subjective and objective criteria for OSAS, using Kotlow’s criteria ^20^ for measuring the lingual frenulum, measuring the free length of the tongue (the distance between the insertion of the lingual frenulum at the base of the tongue and the tip of the tongue), considering the lingual frenulum to be shortened if the length were less than 16 mm. In that study, they concluded that of the 42 children at high risk for OSAS in a subjective assessment, 18 (42.8%) had a shortened lingual frenulum, and the incidence in the study was 114 patients (22.6%). In a multivariate logistic regression model, the risk for OSAS in children with a shortened lingual frenulum was significantly higher than other possible factors, such as obesity, age, gender, and lingual tonus.

In the only prospective study included in this review, Baxter et al.^21^ followed 37 children from 13 months to 12 years of age. For the evaluation of the lingual frenulum, Kotlow’s protocol was followed.^20^ Twenty-four of them were snorers before being submitted to the lingual frenectomy, of which 16 improved the snoring after the surgery. The study concluded that, in addition to improved sleep, there was an improvement in speech and deglutition of solid foods, as reported by parents.

In a retrospective cohort, Zaghi et al.^22^ followed individuals of all ages, including 110 children. The lingual frenulum was assessed using the lingual frenulum and tongue mobility scale,^23^ which includes a comparison of the measurements of maximum mouth opening and opening with the tip of the tongue on the palatine papilla, considering a normal measurement when there is less than 50% of difference between them. The study concluded that lingual frenuloplasty with myofunctional therapy is safe and potentially effective for the treatment of mouth breathing, snoring and myofascial tension.

## Discussion

There is agreement between the four analyzed studies that the lingual frenulum alteration interferes with the growth of anatomical structures, leading to alterations in respiratory, suction, chewing and speech functions. Marchesan^24^ reports that among the alterations caused by an altered lingual frenulum, the most common are those related to speech, nutrition, with special attention to breastfeeding, and deglutition functions. There is a consensus in the literature that stomatognathic functions are affected by alterations in the lingual frenulum.

Considering the results found in the retrospective study by Guilleminault et al., in which two groups were studied, one with a shortened lingual frenulum and the other with a normal one, where significant anatomical differences were observed between the two groups, and the group with the shortened lingual frenulum had a high palate and maxillary atresia, favoring airway collapsibility, a question arises: would early treatment have an effect, improving the symptoms and potentially preventing the onset of a more severe picture of obstructive disorder?

Zaghi et al. published a retrospective cohort study aimed at exploring the safety and efficacy of lingual frenuloplasty and myofunctional therapy in patients with restricted tongue mobility. A total of 348 patients who complained of one or more of the following symptoms were included: mouth breathing (n = 226), snoring (n = 151), dysfunctional deglutition pattern (n = 130), myofascial tightness (n = 44) and/or pain or tension (n = 151) and who were treated with lingual frenuloplasty and myofunctional therapy. Anterior lingual frenectomy was performed in 41 of the patients (11.7%), with complementation elsewhere when persistent restrictions in tongue mobility were identified. A myofunctional therapy protocol was performed one month preoperatively and two months after lingual frenectomy. Pain severity and complications were rated on a 10-point visual analogue scale. Changes in overall health-related quality of life and overall satisfaction were assessed using the Likert Scale. Snoring complaints and sleep quality were also assessed with the abovementioned scales, with no nocturnal monitoring exam being performed. The results obtained were an 87% improvement in quality of life through improved mouth breathing (78.4%), snoring (72.9%), teeth clenching (91.0%) and/or myofascial tension (77.5%). The authors affirm that lingual frenuloplasty associated with myofunctional therapy is safe and potentially effective for the treatment of mouth breathing, snoring and myofascial tension, and further studies are required to identify the ideal candidates for this treatment.

Baxter et al. in 2020 published a prospective study of lingual frenectomy carried out with LightScalpel CO_2_ laser, followed by myofunctional therapy carried out in 37 patients (mean age 4.2 years, ranging from 13 months to 12 years), of which 62% were boys and 38% girls. The degree of lingual mobility restriction was assessed using Kotlow’s classification scale. The study comprised the assessment of speech, eating, sleep, and other related symptoms reported on a yes/no basis. The results observed in the first week were that 76% of the parents reported an improvement in speech, 69% observed an improvement in feeding and 85% observed that their children's sleep improved. Within one month, 89% of parents reported improved speech, 83% observed improved feeding, and 83% witnessed improved sleep. According to the reports, the children slept less frequently in awkward positions (*p* < 0.001), kicked and moved less at night (*p* < 0.001). They slept more soundly (*p* < 0.001), woke up less tired (*p* = 0.002), had less teeth grinding (*p* = 0.002), less mouth breathing (*p* < 0.001), and less snoring (*p* < 0.001). Interestingly, additional changes were observed by parents, as children reported less neck pain (*p* < 0.031), less headaches (*p* = 0.008), less vomiting reflex (*p* = 0.002), less mouth breathing (*p* < 0.001), less reflux (*p* = 0.002), less hyperactivity and inattention (*p* < 0.001), and less constipation (*p* = 0.004). The authors stated that these results should encourage health professionals who treat children with speech, eating and sleep disorders to assess possible tongue mobility restrictions in these patients.

In 2020, Villa et al. carried out a study with the aim of evaluating the presence of a shortened lingual frenulum as a risk factor for the development of sleep-disordered breathing (SDB) in school-age children, whether or not they were snorers. A total of 504 children were included, aged between 6 and 14 years, who were evaluated using a clinical tool used in the detection of children at high risk for SDB, called sleep clinical record (SCR).^25^ The total SCR score is calculated considering nasal, oropharynx, dental, craniofacial and occlusion abnormalities. A total SCR score greater than or equal to 6.5 is considered to be positive and associated with a high risk of OSA, defined as an obstructive apnea/hypopnea index (AHI) >1 episode/h. Of the 504 children evaluated in this study, 42 (8.3%) were at high risk for OSA, as indicated by an SCR score >6.5. Of these children, 18 (42.8%) had a shortened lingual frenulum. Among all 504 children, a shortened lingual frenulum was found in 114 (22.6%). The multivariate logistic regression model showed that children with a shortened lingual frenulum had a significantly higher risk for a positive SCR and, therefore, a greater risk for developing OSA, when compared to those with a normal lingual frenulum and that this higher risk was more significant even when other factors that can possibly affect the risk of OSA (age, gender, tongue strength, obesity) were taken into account. The authors concluded that an association between a shortened lingual frenulum and SDB was demonstrated, and that a multidisciplinary approach is required to allow the early detection and timely treatment of craniofacial changes and potentially prevent SDB.

The studies by Zaghi et al.^22^ and Baxter et al.^21^ showed that the association of myofunctional therapy with lingual frenectomy brought benefits to treated patients, showing the importance of a multidisciplinary action. In a study carried out in 2016 with 101 patients, Ferrés-Amat et al. submitted 96% of patients to the myofunctional therapy, starting one week before performing the lingual frenectomy, aiming at increasing lingual mobility and reducing pain.^26^

The four studies show significance in the relationship between the lingual frenulum alteration and OSA. Further studies with surgical intervention are needed, as well as polysomnographic examinations before and after lingual frenectomy in a larger sample of the population.

## Conclusion

The results of these studies suggest that speech, mastication, breathing and sleep can be affected by a tongue with restricted mobility, and that its adequate surgical release associated with myofunctional therapy can result in functional and quality of life improvement. The improvement in sleep quality was significant in patients included in the studies, in which the intervention was performed, because, after the intervention, the tongue was able to rest on the palate instead of resting on the floor of the mandible. The possibility of tongue elevation, and the releasing of the restriction so that the elevation normalizes, is the reason for the observed improvement.

The studies included in the present review corroborate the association between ankyloglossia and obstructive sleep apnea.

This article is part of the master's thesis of the Surgery Sciences Program at the Faculty of Medical Sciences of the State University of Campinas (UNICAMP).

## Funding

This research did not receive any specific grant from funding agencies in the public, commercial, or not-for-profit sectors.

## Conflicts of interest

The authors declare no conflicts of interest.
